# Elements of Psychological Medicine
*Elements of Psychological Medicine: an Introduction to the Practical Study of Insanity. Adapted for Students and Junior Practitioners. By Daniel Noble, F.R.C.S., Medical Officer to the Clifton Hall Retreat, and Lecturer on Psychological Medicine at the Chatham-street School of Medicine, Manchester. London: John Churchill. 1853.


**Published:** 1854-01-01

**Authors:** 


### Art. II.—ELEMENTS OF PSYCHOLOGICAL MEDICINE.*

An Introduction to the study of Medical Psychology, edited upon the plan of Tennemann's "Manual of the History of Philosophy," is much wanted; we therefore hailed with satisfaction the announcement of Mr. Noble's work, and were induced to regard it as a good sign, indicating the progress of a science which admits of being taught in an elementary form—but we have been disappointed; the book of Mr. Noble does not supply the *desideratum* which exists; and we are moreover bound, in all fairness, to make known that its contents do not correspond with its title-page.

In the high courts of literary criticism, it has always been esteemed a grave offence to put forth any book under a fictitious title; for observe the result! Allured by an attractive title-page, the scholar in pursuit of knowledge orders a copy of the work from his bookseller; it is sent to him, and when too late, he discovers that it is not what he expected. He finds himself in the position of a country cousin, who, upon visiting London, allows himself to be seduced by a gasconading bill of the play into a theatre, where the performance disappoints him; and, we believe, in neither case, according to the law of the Medes and Persians, is there ever any money returned at the doors. Upon this lax principle, we have heard of fashionable publishers occasionally ordering books to be written up to the title—*ce n'est que le premier pas qui coute*—and, unfortunately, the title-page of a book is often its sole recommendation. Speaking generally, men of science, we believe, are obliged to sail a little nearer to the wind. We do not expect to find, under the title of Elements of Mineralogy, speculations on geology, nor under the superscription of Elements of Psychological Medicine did we expect to meet with a series of commonplace lectures on the nature and treatment of insanity. But of this anon! When such books as the present have come before us, we have often wondered what evil genius prompted some men to become authors. It would be a curious revelation, if it were possible, by any psychological analysis, to arrive at the real motives which urge some men to that species of moral suicide, which at once destroys that reputation for learning and ability, which the good-natured over-credulous world is almost ever ready to award to persons who look wise and maintain prudential silence. The portentous shake of Lord Burleigh's head, in

* Elements of Psychological Medicine: an Introduction to the Practical Study of Insanity. Adapted for Students and Junior Practitioners. By Daniel Noble, F.R.C.S., Medical Officer to the Clifton Hall Retreat, and Lecturer on Psychological Medicine at the Chatham-street School of Medicine, Manchester. London: John Churchill. 1853.

"The Critic," carries with it a world of wisdom—it is a fine moral lesson! We can understand an enthusiastic and youthful dramatic poet—snubbed by Garrick in the green-room, and Curl at his office door—rushing into the street, manuscript in hand, exclaiming, "'Sdeath! I'll print it, and shame the fools!" We can enjoy the sly satire of the noble poet.

> "'Tis pleasant, sure, to see one's name in print;
> A book's a book, although there's nothing in't;
> Not that a *title's* sounding *charm* can save
> Or scrawl or scribbler from an equal grave."

We can make every allowance, too, for the foolish vanity which induces some men of imaginary genius to imitate the example of Pygmalion, and fall down in admiration before the creation of their own hands—but why a well-to-do steady-going medical practitioner should step out of his path to make himself conspicuous as an author, when he has nothing new to say on any subject, is to us an inexplicable enigma. The excuse of having been constrained to publish, at the urgent request of many friends, we had supposed an exploded joke; almost as absurd is the apologetic plea set forth in the preface of the volume before us, that it contains the *bonâ fide* substance of a course of lectures delivered at a Neo-Platonic School of Medicine in one of the back streets of Manchester. We see, in plain English, no reason for the publication of a work which does not approach the void in medical literature which it pretends to fill; for instead of being an Introduction to the study of Medical Psychology—or Psychological Medicine (we are indifferent how the words are played upon)—we find the volume split into eight lectures, presenting us with nothing more than a flimsy elementary treatise upon insanity, unworthy of any place upon a shelf near Prichard, Burrows, Morrison, Haslam, Arnold, to say nothing of Esquirol, Pinel, Brierre de Boismont, Georget, and a host of authors upon cerebral and mental diseases, which are at the command of every well educated student. We do not speak with more severity than the occasion warrants—nay, Mr. Noble himself assumes in his preface a tone of such marvellous self-complacency, that we need feel little compunction in taking him to task; he tells us plainly, that if his book fail to realize his little expectations, he may feel "a little disappointment, but certainly no mortification." The soothsayer, it will be remembered, when wounded in the Temple of Apollo, drew the arrow from his side, declaring that it would enable him ever afterwards to deliver oracles. We have, therefore, no scruple in dealing very candidly with so amiable and so self-willing a martyr.

We have declared—and the charge is a serious one—that Mr. Noble's book is not what it professes to be; and in proof of our assertion we now quote the following passage from the preface.

"The author's design, in the present work, has been to treat only of what is commonly understood by insanity, or mental derangement—the condition, in fact, so recognised by law and custom—in such a manner as to introduce the topic in a scientific form to those who have previously given it no special consideration." (p. xiii.)

Why not fairly, therefore, give the book a title corresponding with this design? The title-page, as it stands, has, we have reason to know, deceived many persons, who naturally expected to find the elements of Psychological Medicine here set forth in a way somewhat similar, perhaps, to that which has been so well accomplished by Feuchtersleben, in his Principles of Medical Psychology. The substantive words Psychology and Insanity are clearly not synonymes; in point of fact, medical psychology embraces a much wider field than comes within the range of insanity, inasmuch as it comprehends the study of all mental phenomena, whether normal or abnormal; while the word Insanity, applied as it may be to all the different forms of mental derangement, has a much more restricted meaning. But this is not all. When a new work is announced as containing the "Elements" of any given science, we naturally expect to find therein the rudimentary principles and facts upon which such science has been founded. All university text-books are compiled expressly with this view; but in the present case, Mr. Noble's "Elements of Psychological Medicine" is a book which does not supply the student with the slightest elementary knowledge of medical psychology. He sets out with defining "Psychology" truly as a word of Greek derivation, signifying a discourse on the soul; but this done, he starts off at a tangent, and very ungratefully drops the adjective which he has so prominently emblazoned on his title-page. Nay, more: in dealing with Insanity, he plunges at once *in medias res;*—nor can we discover, through the hazy light of his first introductory lecture, that he gives his pupils any elementary knowledge whatever—not the slightest clue to guide them through the labyrinth into which he introduces them. He tells them that insanity, as all are aware, is the generic term which comprehends the class of mental diseases; and he adds—"I very much doubt if any definition that can be given will suggest to the student a clearer understanding of the subject than does the term itself." This certainly is cutting the Gordian knot of a difficulty which has puzzled many learned authors. The students of the Chatham-street School of Medicine, we presume, forthwith closed their Lexicons, fully satisfied that the etymology of a word is not likely to throw any light upon its signification or proper use. We beg to call the attention of the Philological Society to this important discovery, which may very materially abridge many of their curious speculations.

"In almost every department of knowledge," continues our recondite lecturer, "definitions have been given that utterly defeat the intention of all definition, which is to render the subject more intelligible than it was before; and this is remarkably so in the matter of insanity."

We are by no means certain that this definition of a definition renders the meaning of that word a whit clearer; but this much is evident, that if the word Insanity be so expressive in itself as to supersede the necessity of any further definition, the author need not have hazarded a definition professedly of his own, which appears to us the very worst we ever met with, inasmuch as it is a definition which does not comply with the logical requirements of a definition, and contains as many blunders as it does words. "For convenience," says the lecturer, "and with reference to the views which I shall advance in the course of these lectures, I *will venture upon a brief definition of my own, and will define Insanity to consist in chronic disorder of the brain, inducing perversion of ideas prejudicial to, or destructive of, the freedom of the will.*" We had always supposed that a definition aimed at explaining a thing by its essential attributes—those which are common (*genus*) and those which are proper (*difference*)—and that three things were considered necessary to a good definition—viz., that it should be universal, that it should be appropriate, and that it should be clear; instead of complying with which several conditions, the definition before us is a jumble of unintelligible theories, and, in point of fact, it has no pretension to being even called a definition. This the author seems himself conscious of; for he adds, very coolly—"*Of course, it may be objected to this definition that it defines nothing—that every part of it requires itself to be defined.*" (p. xxi.) Why, if it define nothing, then put it forth as a definition? Why tamper with the understanding of the intelligent audience which doubtless listened with profound admiration to these lectures? But what follows? "I am well aware," continues Mr. Noble, "that numerous questions underlie the *category* which I have advanced apparently so simple." (Ibid.) So then, by a curious kind of metaphysical harlequinade, the *category* becomes the *definition*, and the *definition* the *category!* It is evident enough that the learned author never puzzled himself in studying the Organon of Aristotle, or the dialectics of the Cartesian, Wolfian, or Kantian philosophy; but we venture to hint, that even from humbler sources, it would be useful for a public lecturer to ascertain, and bear in mind, the meaning of the elementary terms which are employed by all logicians in reasoning upon philosophical subjects. To return, however, to the so-called definition: what does Mr. Noble, in a physiological or pathological point of view, mean by defining Insanity as consisting in chronic disorder of the brain? Has Mr. Noble, in the course of his domiciliary visits at Clifton Hall Retreat, never met with a case of acute mania?—and if so, has he not observed the rapidity of the pulse—the hurried breathing—the flushed countenance—the wild glistening of the eyes—the fierce expression—the contracted forehead—the corrugated eyebrows—the throbbing at the temples—the burning scalp—and the intense exaltation of all the sensorial functions? Has he not noticed the lightning-like rapidity with which ideas rush apparently through the brain, unconnected with each other, giving rise to that strange confusion and incoherence of thought and feeling which has been perhaps as finely described by Coleridge, in his "Pains of Sleep," as by any medical author.

"A lurid light, a trampling throng,
Sense of intolerable wrong,
And whom I feared, those only strong!
Thirst of revenge—the powerless will
Still baffled and yet burning still!
*Desire with loathing strangely mixed,*
*On wild or hateful objects fixed;*
*Fantastic Passion's maddening brawl,*
*And shame and terror over all;*
*Deeds to be hid which were not hid;*
*Which all confused, I could not know*
*Whether I suffered or I did.*"

This state of mental anarchy, this confusion of thought and feeling, perplexing and bewildering consciousness itself, we can understand, when we consider that the consecutive relation between ideas is destroyed by excessive cerebral excitement; and it cannot be disputed, for it has been proved by ocular demonstration, that in all such cases there is an increased flow of arterial blood to the brain, which lights up an inflammatory action, and this we find constantly taking place in the incipient stages of insanity.

Or we may venture to ask Mr. Noble if he has never attended the post-mortem examination of persons who have died from an attack of acute mania?—and if so, whether he has not observed the brain and its membranes presenting all the signs of recent acute inflammation; prodigious congestion of the superficial bloodvessels; the pia mater and arachnoid membranes highly injected; adhesions between the convolutions; the grey matter of the brain preternaturally vascular; minute extravasations and bloody points dispersed through the substance of the hemisphere; ventricular effusion, and other signs, indicating plainly that here we have had *acute*, not *chronic* disease, disturbing the normal relation between the mind and its material organ. We readily grant that we have, in many cases, insanity obviously depending upon chronic disease of the brain—our public asylums are unhappily crowded with such cases; but in its incipient stages the disease constantly assumes an *acute* form; we therefore emphatically repudiate the proposition (we will not call it a definition) that "insanity consists in chronic disorder of the brain."

Nor is Mr. Noble more fortunate in describing insanity as consisting in "perversion of ideas prejudicial to, or destructive of, the freedom of the will." All misstatements and blunders may be more or less entangled with a certain amount of truth; thus, in some cases of insanity, false perceptions may exist from some obscure lesion of the organs of sense or morbid condition of the brain; hence a variety of visual or aural illusions may arise, which will communicate false impressions to the mind; as when the notable knight of La Mancha mistook a flock of sheep for an army, and a windmill for a giant. There may also, in some cases—as in impulsive insanity—exist a perversion of ideas, accompanied by an irresistible desire to commit some insane act, which the will, as the controlling faculty of the mind, cannot restrain. But, on the other hand, the perceptions of the insane are, in other instances, marvellously clear, and they will not unfrequently reason upon a variety of subjects with almost preternatural lucidity. It by no means follows, therefore, that we shall find, in all cases of insanity, a perversion of ideas, or a lesion of the power of volition; indeed the will of the insane, so far from being paralyzed or destroyed, frequently evinces a remarkable power of self-sustaining energy. The will of the insane is often, too, as much under their command as the will of the sane; and the perseverance and intensity with which it effects its purpose greater in the former than in the latter. Look at the prodigious perseverance a lunatic will take to carry out any wild scheme. With a rusty nail, he will, to effect his escape, for weeks and months work day and night, striving to disintegrate a stone wall; and when not so occupied, affect a perfect indifference respecting his detention, and evince a conscious self-command over all his actions. The indomitable will of the insane (however misdirected) must be familiar to all who have had any experience in lunacy; indeed, we apprehend that no satisfactory definition of insanity can be based upon the lesion or aberration of any one mental faculty. We know that Cullen ascribed the disease to false perception of external objects, giving rise to erroneous judgment; Battie and Ferriar conceived that false perceptions gave rise to confusion of ideas; while Mason Good, following Locke, argued that the judgment was principally at fault; but from the views which psychologists have recently adopted, we are led to regard the mind as a perfect unity. "To conceive of mind," says Morell, "under the idea of a multiplicity of powers and operations, will always, in the long run, prove untenable. We know that it is one. The unity of consciousness is at once the deepest, surest fact of our nature, and the most rigid condition for a complete mental philosophy."* Perception, attention, memory, judgment, volition, imagination, may all be more or less implicated in the disease; but we cannot dissect out either of these faculties from the mind, as we might the different parts of a bodily organ, and say it is the lesion of this or of that particular faculty which constitutes the true pathology of the disease. Hence the failure which has attended the definitions of insanity, proposed by so many eminent medical authorities, upon psychological distinctions which are purely arbitrary. Hence, strange as it may appear, unprofessional authors, being, it may be presumed, unshackled by physiological or pathological theories, and those observers of nature who have aimed rather at giving a *description* than a *definition* of insanity, have succeeded better than some of our best nosologists, not excepting Cullen, Sauvages, Vogel, or Mason Good. We cannot afford space for the digression, or we might illustrate the truth of this observation by extracts from many popular writers; we may, however, be permitted to quote the following passage, which will be met with in Hartley Coleridge's very charming essay on the character of Hamlet: "To be mad is not to be subject to the common laws whereby mankind are held together in community; and whatever part of man's nature is thus dissociated is justly accounted insane. If a man see objects or hear sounds which others, in the same situation, cannot see or hear, and his mind and will assent to the illusion (for it is possible that the judgment may discredit the false intelligence), such man is properly said to be out of his senses, though his actions and conclusions from his own peculiar perceptions should be perfectly sane and rational."† It was Percy Bysshe Shelley if we remember right, who condensed this view pithily in a single line—

"And he was mad—*if madness 'tis to be unlike the world.*"

But the true cause of the difficulty of giving a successful definition in any science, Professor Whewell has clearly pointed out to consist in this—that there must be a clear conception of the nature of the thing to be defined; it must be thoroughly understood in all its relations before it can be formally expressed; for which reason, writers on logic in the middle ages made definition the last stage in the progress of knowledge. We do not marvel, therefore, that Mr. Noble should have failed in fixing upon a definition which, albeit enunciated as his own, and impressed with the seal of his originality, is nevertheless an almost literal travesty of a passage in Dr. Prichard's "Treatise of Insanity," where we find, at p. 7, insanity described as a "chronic disease," manifested by "perversion of the feelings." By substituting the word

* "Elements of Psychology," p. 18.
† "Essays and Marginalia," vol. i. p. 163.

*ideas* for *feelings*, and involving clumsily the freedom of the will, Mr. Noble appears to have arrived at the above apocryphal definition. We do not accuse Mr. Noble of open, barefaced, plagiarism—amounting to what Dogberry would call "flat burglary as ever was committed"—but there is, *certes*, a very suspicious family likeness between the parallel passages.

The hypothesis which Mr. Noble's theory involves, that insanity consists in a *chronic* disorder of the brain, necessarily vitiates, *in limine*, his *ex cathedra* instructions upon the nature, treatment, diagnosis, and pathology of the disease. Acute mania is necessarily excluded from his nosography, and the ordinary classification of insanity superseded by one of his own—as original, by the way, as the above definition. All writers and statistical records, Mr. Noble tells us, "have their cases of mania, melancholia, and dementia; and other familiar terms are constantly employed. These, however, do but exhibit the more salient groups of the pathological picture; and in many instances they have little more fixedness than so many dissolving views. For a case of melancholia may become one of mania; or the two affections may be present simultaneously" (p. 124). This is obviously very superficial reasoning. It is quite true that a state of mania may succeed to a state of melancholia, and *vice versâ*, or the two forms of the disease may occasionally appear to be blended; but, notwithstanding this, the type of mania and the type of melancholia has each its specific characteristics. Disease of the heart may supervene upon phthisis, or both terminate in dropsy, which may co-exist with either disease; but nosologists describe each as a different disease. So also mania may subside into melancholia, melancholia into dementia, and dementia into idiocy, but each of these different forms of insanity being characterized by specific features, lecturers very properly describe them separately for the instruction of their pupils. Above half a century ago, the learned Dr. Arnold proposed subdividing insanity into ideal insanity (the intelligential of Mr. Noble), notional insanity, and appetitive insanity (which corresponded with our descriptions of emotional insanity). This arrangement was rigorously criticised by Dr. Alexander Crichton, in his "Inquiry into the Nature and Origin of Mental Derangement," and did not maintain its ground in our medical schools, because the classification is obviously founded upon psychological distinctions, which are so much less obvious than the physical signs which induced Esquirol and Pinel to adopt the classification which Mr. Noble regards in the light "only of dissolving views." Under the head of notional insanity he includes monomania; and under that of intelligential (*ideal*) insanity, idiocy, dementia, and mania. We may observe, *en passant*, that Mr. Noble makes no allusion to this classification having been originally suggested by Dr. Arnold; in fact, throughout these lectures he does not refer his pupils to a single authority upon medical psychology, which would doubtless have moved the wrath of honest old Burton, who tells us, in his "Anatomy of Melancholy," that he cites and quotes his authorities, because he holds, with Synesius, that it is a greater offence to steal dead men's labours than their clothes—*Magis impium mortuorum lucubrationes quam vestes furari!*

To return. The views of Mr. Noble respecting his subdivisions of intelligential insanity appear to be not a little mystified; he tells us that "the *conventional* terms, *idiocy* and *dementia*, will express the *negation* and the deterioration of the intellect" (p. 141); and shortly afterwards he states that "dementia in its actual phenomena is *identical* in a great measure with *idiocy;* in both conditions the essential feature consists in the absence or notable diminution of intelligential power" (p. 150). Here again we are at issue with the learned lecturer, inasmuch as the word dementia does not express a state of mental negation, neither is it in any measure identical with the word idiocy. What is the approved meaning of this word, dementia, as propounded by Pinel? It is that form of insanity, says this eminent authority, which is characterized by "rapid succession or alternation of insulated ideas, and evanescent and unconnected emotions, continually repeated acts of extravagance, complete forgetfulness of every previous state, diminished sensibility to external impressions, abolition of the faculty of judgment, perpetual activity." In dementia we have, as here described, a host of incongruous ideas busily passing through the mind; but idiocy is a state of mental negation presenting us with the very opposite condition, for from some natural defect of the understanding consequent upon imperfect organization, the mind of the congenital idiot is, as Locke has well described, incapable of receiving or retaining any ideas. If Mr. Noble had ever observed the phenomena of acute dementia, assuredly he never would have described it as a form of disease identical with idiocy. In the next page, it is true, he draws a shadowy diagnosis between these two mental states; still he fails to give his pupils anything like a correct description of either mental condition. He is not much more fortunate with his description of mania, which he illustrates by a rambling letter addressed to himself by a patient at Clifton Hall.

In each lecture Mr. Noble attempts a variety of pathological observations, all of which are extremely vague, and amount literally to nothing; indeed, nearly at the end of his course, at the beginning of the fifth lecture, we were taken with surprise by the following declaration:—

"In the varieties of mental derangement described in the last lecture, in the cases wherein the presence either of normal illusions or of marked disturbance of the intelligence constitutes the leading characteristic, we are led by considerations both physiological and pathological to regard the hemispherical ganglia as the special site of the ailment. But whether the several modifications which these varieties offer involve distinct portions of the grey matter of the convolutions, or consist rather in some difference of pathological alteration which the affected structure undergoes, I feel it impossible to say. *I am unacquainted with any facts capable of throwing satisfactory light upon the subject, and unprepared with any speculations tending to elucidate it.*" (p. 165.)

In a state of such profound and confessedly hopeless ignorance, "unacquainted with *any* facts,"—"unprepared with *any* speculations," we not only marvel that Mr. Noble should have had the temerity to deliver this course of lectures, but that he should afterwards have presumed to publish them as setting forth the "Elements of Psychological Medicine." Before assuming the professor's toga, Mr. Noble ought to have better qualified himself for the discharge of his duties; he ought to have "read up" for the occasion, as lawyers do when they get up special cases, and he should have taken care to have had a more elaborate brief before him; for it was obviously Mr. Noble's duty to obtain such information as might enable him to lay before his pupils the facts which are known respecting cerebral pathology in connexion with mental disease. The suicidal confession of his being unacquainted with any, recoils upon himself, and would imply that he is wholly ignorant of the researches and observations of Foville, Parchappe, Guislain, Brierre de Boismont, and a host of French pathologists; nay, if Mr. Noble had only given himself the pains to examine the *Annales Psychologiques*, or—and we affirm this without exposing ourselves to the least charge of vanity—if he had consulted many of the numbers of our own Journal, he might have alighted upon some facts which would perhaps have materially dispelled the darkness of which he complains. To allege that no progress has been made in the pathology of the brain, either abroad or at home, and that no facts exist which throw light on the structural changes which take place in certain mental diseases, is simply absurd, and would suggest the propriety of the lecturer changing places with one of his more intelligent pupils, for he evidently wants the information which he professes to teach. In such a position we can readily understand that Mr. Noble was, as he states, "unprepared with any speculations tending to elucidate the subject," for theories are suggested by facts, and Mr. Noble avows that he has none at his command. In such an extremity, therefore, he could not, like Mephistophiles guiding his scholar Faustus through the Brocken, even hail a single *ignis fatuus* to cheer his benighted class.

In the course of these Lectures, Mr. Noble formally announces to the scientific world that he has ceased to be a phrenologist. "The system of phrenology," he says, "*cannot*, I am convinced, be sustained by a just philosophy." We may here, perhaps, be permitted a slight digression, for "thereby hangs a tale." In the year 1846, Mr. Noble published a goodly octavo volume, entitled "The Brain and its Physiology," which is, from page 1 to page 450, written avowedly upon phrenological principles; and therein he goes out of his path to revive a controversy which, by his own confession, had been disposed of about sixteen or seventeen years ago."* The facts we believe to be briefly these: The late Dr. Milligan—the learned translator of Celsus—in the Appendix to his Translation of Magendie's "Physiology," suggested that instead of bringing the light artillery of wit and ridicule to bear upon the system, or fighting upon obscure metaphysical grounds, a direct *experimentum crucis* should be made, and different heads and characters compared with each other. The celebrated Sir William Hamilton, now Professor of Logic in the University of Edinburgh, thereupon instituted a very interesting, and, at that time, a very curious, series of experiments; and after weighing brains, measuring crania, &c., he came to the conclusion that not only was phrenology untrue, but that its several propositions led to the very antipodes of truth. This induction was ably followed up by Dr. Stone; thereupon Mr. Combe buckled on his armour, and then a fierce Junius-like controversy took place, and we can scarcely remember which party came off victorious; but, sixteen or seventeen years after this, it was not a little ludicrous to find Mr. Noble taking the field in a Hudibrastic fashion, to again and again slay the already slain. The attack which Mr. Noble made on Sir W. Hamilton and his contemporaries was, of course, highly successful! How could it be otherwise? He had no enemy to contend with; the field had been already left; the victory therefore rested, as the book on the Brain before us assumes, with Mr. Noble; so have we seen, in our boyhood days, a victorious army sweep across the stage of Astley's Theatre, followed at a distance by Mr. Merryman on an ambling nag, blowing a penny trumpet, and congratulating himself upon the victory. But mark what follows! Seven years have now elapsed—the true Pythagorean period for the regeneration of fallen humanity—and behold Mr. Noble comes out in the Lectures before us a veritable anti-phrenologist—nay, takes up the very arguments (of course, without acknowledgment) which were formerly used by the men whom he assailed. He tells us that his "doubts of the validity of phrenology were first occasioned by the perusal of an article by Dr. Carpenter, in the "British and Foreign Review" (Preface, p. x.); and in his "Lecture on the Physiology of the Brain and Nervous System" (pp. 36—83), he details the reasons for his secession—or, in more fashionable language, for his "perversion"—from the true Church of

* Noble on the Brain, p. 279.

Phrenology, with as much circumstantial detail as if it were an event of as great importance to the world as the conversion of Constantine to Christianity, or the apostasy of Julian. We apprehend, however, Mr. Noble's apostasy from phrenology will give the friends of that science as little concern as his attacks gave the anti-phrenologists, who, we believe, never took the least notice of them. He will now, we fear, be respected by neither party, thus illustrating the truth of the old proverb, propounded by no less a philosopher than Seneca—*inter duas sellas decidium.*

We repeat, in conclusion, that Mr. Noble's book, addressed to those who are engaged in the practical study of insanity, will not supply them with the information it professes to give; it is, *de facto,* not a work on the Elements of Psychological Medicine; and we feel fully justified in endorsing the volume before us with the Virgilian caution, "*Nimium ne crede colori,*" which we may freely translate—"Never buy a book from its title-page!"

---

